# Extending the Applicability of the Multiple-Spawning Framework for Nonadiabatic Molecular Dynamics

**DOI:** 10.1021/acs.jpclett.2c03295

**Published:** 2022-12-21

**Authors:** Yorick Lassmann, Daniel Hollas, Basile F. E. Curchod

**Affiliations:** Centre for Computational Chemistry, School of Chemistry, University of Bristol, BristolBS8 1TS, U.K.

## Abstract

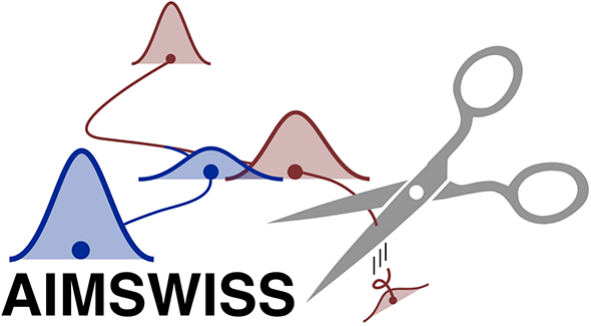

*Ab initio* multiple-spawning (AIMS) describes the nonadiabatic dynamics of molecules by expanding nuclear wave functions in a basis of traveling multidimensional Gaussians called trajectory basis functions (TBFs). New TBFs can be spawned whenever nuclear amplitude is transferred between electronic states due to nonadiabatic transitions. While the adaptive size of the TBF basis grants AIMS its characteristic accuracy in describing nonadiabatic processes, it also leads to a fast and uncontrolled growth of the number of TBFs, penalizing computational efficiency. A different flavor of AIMS, called AIMS with informed stochastic selections (AIMSWISS), has recently been proposed to reduce the number of TBFs dramatically. Herein, we test the performance of AIMSWISS for a series of challenging nonadiabatic processes—photodynamics of two-dimensional model systems, 1,2-dithiane and chromium (0) hexacarbonyl—and show that this method is robust and extends the range of molecular systems that can be simulated within the multiple-spawning framework.

Following their interaction with light, molecules will in the course of the ensuing dynamics explore regions of nuclear configuration space in which the main assumptions of the Born–Oppenheimer approximation^1,2^ are no longer valid. To simulate the resulting nonadiabatic dynamics, numerous computational methods have been developed in the past decades.^3^ Numerically solving the time-dependent Schrödinger equation on a grid is the most accurate approach but suffers from exponential scaling with respect to dimensionality of the system. Despite extensive development of grid-based methods, such as multiconfigurational time-dependent Hartree (MCTDH)^4,5^ to circumvent this problem,^6−8^ many methods have instead been developed around the idea of using a moving basis set of *coupled* frozen Gaussians,^9,10^ also known as trajectory basis functions (TBFs). Examples of such methods include variational multiconfigurational Gaussian (vMCG)^11−13^ that propagates TBFs variationally, multiconfigurational Ehrenfest (MCE)^14−16^ for which TBFs evolve on mean-field potentials, and *ab initio* multiple-spawning (AIMS),^17−21^ which propagates TBFs classically on individual adiabatic potential energy surfaces (PESs). More recent work within the TBF-based framework has focused on finding alternatives to the adiabatic representation of electronic states, alleviating some of its shortcomings.^22−25^

A hallmark of the AIMS method is its titular spawning algorithm, which is how AIMS allows its basis of TBFs to grow whenever multiple electronic states become close in energy and nonadiabatic transitions are likely to occur.^20,26^ Within the spawning algorithm, a parent TBF—the TBF that encounters significant nonadiabatic coupling with other electronic states—will make copies of itself onto these coupled states, thus creating child TBFs. Afterward, the coupled dynamics is continued within this extended basis set. Even though the adaptive nature of the basis results in a high accuracy for AIMS, it is a double-edged sword. A notorious issue with the spawning algorithm occurs when multiple crossings between electronic states happen during a nonadiabatic dynamics. In such a scenario, the basis set can grow uncontrollably to the detriment of computational efficiency. An approach to thwart such unrestrained growth and systematically reduce the number of TBFs was recently introduced and coined stochastic-selection AIMS (SSAIMS).^27^ This method is based on the realization that groups of TBFs that are far away in phase space will only be weakly coupled and can thus be propagated independently. Hence, as soon as groups of TBFs separate in phase space, SSAIMS randomly selects one of them based on their nuclear population to carry on the dynamics and removes all other TBFs.

While it dramatically increases the computational efficiency of the multiple-spawning framework,^28^ SSAIMS introduces a new parameter—the selection threshold—which allows the method to distinguish between coupled and uncoupled (groups of) TBFs. This parameter monitors either the Hamiltonian matrix element between TBFs (ESSAIMS) or their overlap (OSSAIMS). Since the optimal selection threshold has to be determined for every new molecule under study, the gain in efficiency offered by SSAIMS may only be apparent. To solve this problem, we have recently developed the AIMS with informed stochastic selections (AIMSWISS)^29^ method that replaces the fixed selection threshold with an adaptable parameter, which we shall discuss in more detail below. We have shown that AIMSWISS can accurately reproduce the nonadiabatic dynamics obtained from SSAIMS and AIMS while being less computationally costly than these techniques. This original work constitutes mainly a proof of principle, though, as AIMSWISS was only tested on a limited set of rather simple organic molecules. In this Letter, we wish to reveal the inner workings and capabilities of the AIMSWISS method by testing it on a selected set of molecular systems and models displaying challenging photodynamics: (*i*) a collection of three 2D model systems offering an opportunity to analyze the underlying assumptions of the AIMSWISS algorithm for different types of conical intersection; (*ii*) 1,2-dithiane, which exhibits a long-lived degeneracy between three electronic states upon photoexcitation; (*iii*) the organometallic complex [Cr(CO)_6_], chromium (0) hexacarbonyl, whose size and density of electronic states push the boundaries of the original multiple-spawning framework. We will show in the following that AIMSWISS strikes a good balance between efficiency and accuracy, reproducing in all cases the results of AIMS while being significantly cheaper for all simulations conducted. In addition, AIMSWISS can warn the user on-the-fly during the dynamics when its assumptions are challenged and a long coupling between TBFs would be required, positioning this method as an ideal starting point within a *hierarchy* of (SS)AIMS methods.

Let us start by presenting how AIMSWISS can predict the loss of coupling between two TBFs. In the spawning algorithm, the phase space centers of parent and child TBFs are nearly identical at the exact spawning time—their positions are the same, while their momenta differ sightly due to rescaling to ensure total classical energy conservation. Based on this observation, it is possible to build upon earlier work by Schwartz and co-workers on the overlap decay of TBFs with identical initial conditions and moving on different adiabatic PESs.^30^ Within a short time limit, one can show that the overlap of the parent-child TBF pair approximately decays as a Gaussian in time,^29^ with a characteristic decay time given by
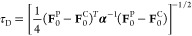
1**F**_0_^P^ and **F**_0_^C^ are the nuclear gradients calculated at the spawning time acting on the centers of the parent (P) and child (C) TBFs. **α** is a diagonal matrix containing the widths shared by all TBFs. We have previously shown that the Gaussian overlap decay model agrees well with the decay actually observed in AIMS and that when there are deviations the model tends to *overestimate* the AIMS decay time.^29^ For this reason, AIMSWISS employs the decay time τ_D_ given by eq 1 to estimate when to perform the stochastic selection of TBFs.

With this background in mind, we can now turn to the individual steps of an exemplary AIMSWISS run (see Figure 1 for a schematic representation). An initial TBF evolves on its assigned adiabatic electronic state and can detect when it approaches a region of nonadiabaticity, triggering a “spawning mode”. In this spawning mode, the coupled propagation of the TBFs is suspended, and the parent TBF will locate the position of a maximum of the nonadiabatic coupling with another electronic state along its trajectory. The parent TBF will spawn a child TBF with zero amplitude on this coupled electronic state at the location of the maximum of the nonadiabatic coupling. Subsequently, the child TBF is back-propagated until the time when the parent TBF entered the spawning mode (for a detailed discussion of the spawning algorithm, please refer to ref (20)). The full coupled dynamics is then reinitialized with the addition of a new child TBF with zero amplitude. This step is depicted in Figure 1, with the child TBF (orange) starting its dynamics with zero amplitude (flat orange line). Thanks to the deterministic nature of the TBF dynamics, the parent and child TBFs will meet at the maximum of nonadiabatic coupling, where they perfectly overlap in configuration space. At this point, one calculates the decay time τ_D_ based on eq 1 using the nuclear forces on the parent (**F**_0_^P^) and child (**F**_0_^C^) at this specific time. The coupled dynamics of the parent and child TBFs carries on for a time τ_D_, when a stochastic selection is performed and one of the TBFs is chosen randomly—based on the nuclear population it carries—to continue the dynamics while the other is discarded from the simulation. The nuclear amplitude of the remaining TBF is renormalized, and the dynamics is continued. In most practical cases, the child or parent TBFs may have spawned further TBFs within the time τ_D_. In this situation, AIMSWISS assumes that the two groups of TBFs—the one comprising the child TBF and its progeny, and the other one containing the parent TBF and its other offspring—will become disconnected after the time τ_D_ has elapsed and one of the groups will be selected. To converge the Monte Carlo algorithm, and consequently to ensure that no valuable information is lost through the removal of TBFs via the stochastic selection, AIMSWISS runs starting from the same initial condition (nuclear positions and momenta) need to be repeated multiple times using a different seed for the random-number generator.

**Figure 1:**
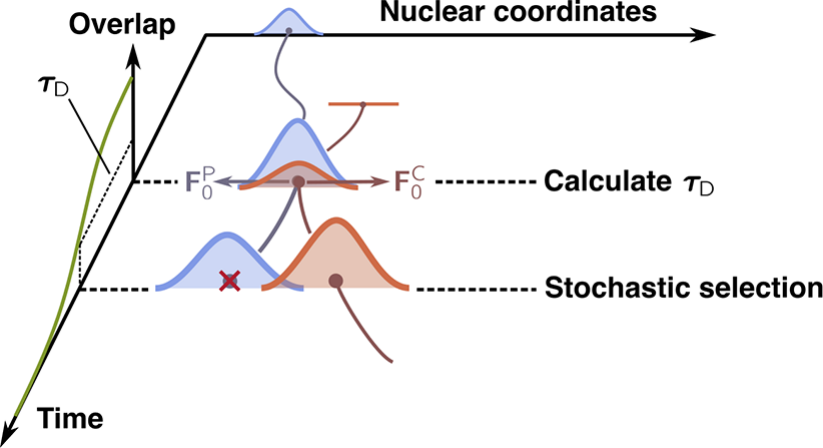
Schematic representation of the AIMSWISS approach. The initial parent TBF is given by a light blue Gaussian and its child TBF by an orange Gaussian. The height of the Gaussians represents their respective population. The overlap between the parent-child TBF pair is represented by the green curve. Note that the child TBF has zero amplitude when it enters the simulation and has a perfect overlap with the parent TBF in configuration space when τ_D_ is calculated. See the main text for a detailed description of the individual steps of the AIMSWISS strategy.

Since the decay time τ_D_ depends on the difference of nuclear forces deriving from two distinct electronic states, one may wonder how the topology of their conical intersection (CI) and the topography in its vicinity affect the result and efficiency of the AIMSWISS method. To answer this question, we make use of a set of two-dimensional, two-state linear vibronic coupling (LVC) models that were first introduced by Ryabinkin et al.^31^ and recently used to study the influence of including the geometric phase and other correction terms on the AIMS dynamics.^32^ These models were constructed to represent the PESs and CI of the molecules bis(methylene)adamantyl cation (BMA), butatriene cation, and pyrazine (see inset of Figure 2d–f for schematic representations). Three different types of CIs are depicted by the models: a seam of intersection (BMA), a moderately sloped CI (butatriene cation), and a strongly sloped one (pyrazine). The nonadiabatic dynamics of a nuclear wavepacket initiated on the S_1_ PES was simulated using AIMS, AIMSWISS, and numerically exact quantum dynamics (QD, using a split-operator formalism^33^ within the diabatic basis). AIMS and AIMSWISS used the same set of 2000 initial conditions, and their nuclear dynamics was performed using a modified version of the FMS90 code implemented in MOLPRO.^34^ The computational details are presented in the Supporting Information (SI).

**Figure 2:**
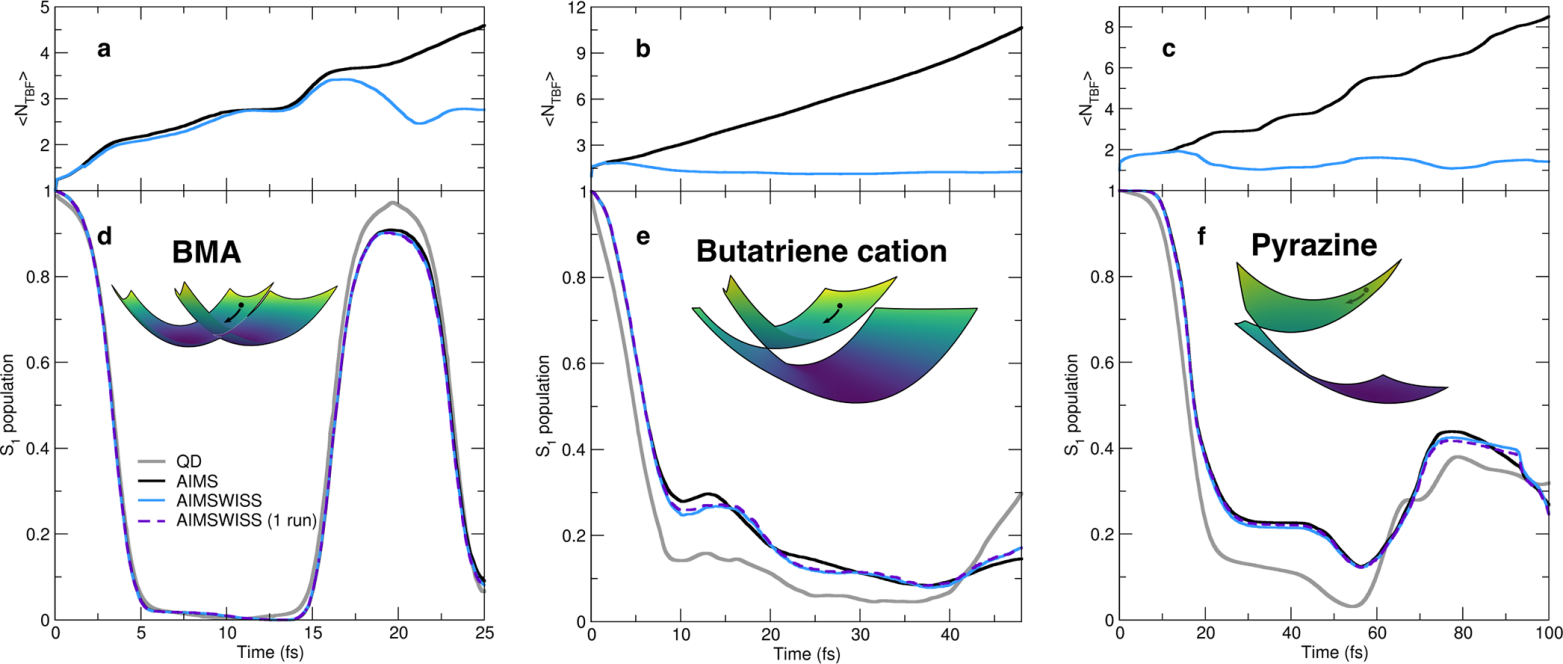
Nonadiabatic dynamics for three reduced-dimensionality models representing the molecules BMA, butatriene cation, and pyrazine. (a–c) Average number of TBFs per time step (⟨*N*_TBF_⟩) for AIMS and AIMSWISS. (d–f) S_1_ population traces calculated with different nonadiabatic dynamics methods: QD (thick gray curves), AIMS (thick black curves), and AIMSWISS – converged (light blue curves) and with only one run per initial condition (dashed violet curves). Inset shows a schematic of the (adiabatic) potential energy surfaces near the conical intersection, with a circle and arrow symbolizing the direction of the nuclear wavepacket in the early stage of the dynamics.

The AIMS and AIMSWISS S_1_ population traces are practically indistinguishable for the BMA molecule (see Figure 2d) and only slightly deviate from the QD reference at ∼20 fs. AIMSWISS also reproduces well the S_1_ population trace obtained with AIMS for the pyrazine model, while for the butatriene cation, AIMSWISS and AIMS differ ever so slightly (<5%) at around 10, 25, and 45 fs. We note that the deviation observed between AIMS/AIMSWISS and the QD reference has previously^32^ been attributed to the limitation of the independent first generation approximation inherent in AIMS.^35^ Interestingly, using AIMSWISS with only one run per initial condition (dashed violet curve in Figure 2d–f) produces an almost identical excited-state population trace to the one obtained from the fully converged AIMSWISS (with three runs for each initial condition) for all three model systems. Thus, converging the AIMSWISS dynamics with respect to the number of initial conditions appears to outweigh the importance of converging the stochastic algorithm.

The average number of TBFs per time step (⟨*N*_TBF_⟩, Figure 2a–c) reveals differences in the efficiency of the selection procedure operated by AIMSWISS for the models studied. AIMSWISS efficiently prevents the growth of the number of TBFs after the initial S_1_ population decay for the butatriene cation and pyrazine models (see the divergence of the AIMS and AIMSWISS ⟨*N*_TBF_⟩ curves in Figure 2b,c). The selection process built into AIMSWISS behaves differently for the BMA model (Figure 2a). The average number of TBFs in AIMSWISS remains close to the AIMS one until long after the first nonadiabatic event (Figure 2a). In other words, the predicted τ_D_ for this model is too large to operate an efficient selection of the TBFs in AIMSWISS following the first nonadiabatic decay. Subjecting the three models to a more detailed analysis (see SI) reveals that the decay time predicted for the BMA model is around 15 fs for most parent-child TBF pairs, while τ_D_ is predicted to be ∼2 fs on average for the butatriene cation and pyrazine models. The strong variation in the decay times between these two types of models is due to the shape and topology of their CI. Whereas the degeneracy between the two electronic states in the butatriene cation and pyrazine models is lifted to a similar degree in all directions of the branching space, the degeneracy in the BMA model is only weakly lifted along the seam of intersection, leading to almost parallel PESs (i.e., similar nuclear forces from the two coupled PESs) in this region. For this reason, the predicted decay time τ_D_ overestimates the actual overlap decay time of AIMS by a factor of ∼10 for the vast majority (∼95%) of parent-child TBF pairs created during the dynamics of BMA. In contrast, overestimations of the actual AIMS overlap decay by τ_D_ are rare in the butatriene cation and pyrazine models (2% for the butatriene cation and 0% for the pyrazine model) because of the efficient lifting of the electronic degeneracy. It is important to note that keeping the parent-child TBF pairs coupled for longer only has an effect on the efficiency of the AIMSWISS method but will have no bearing on its accuracy. Hence, the results obtained for the 2D models show that the nonadiabatic dynamics obtained with AIMSWISS is in close agreement with that of AIMS despite the significant reduction in the number of TBFs propagated. This observation is explained by the fact that, for τ_D_ ≥ τ_AIMS_, AIMSWISS performs stochastic-selection processes when TBFs are only weakly coupled, while the nonadiabatic transitions are still described with an AIMS accuracy.

Conversely to the previous set of calculations, what would happen if the model decay time *underestimates* the actual AIMS overlap decay between TBFs? To answer this question, we shall focus on the photodynamics of 1,2-dithiane simulated with AIMS, OSSAIMS (with a selection threshold for the overlap of ε = 0.5), and AIMSWISS. The same set of 14 initial conditions was used for the three multiple-spawning methods, with five runs per initial condition for OSSAIMS and AIMSWISS. The electronic-structure quantities necessary for the dynamics were calculated at the SA3-CASSCF(6/4) (state-averaged complete active space self-consistent field^36,37^) level of theory using the GPU-accelerated TeraChem^38−42^ software package, and the nuclear dynamics was performed with a version of FMS90 interfaced with TeraChem.^43^ The reader is referred to the SI for the full computational details, together with a discussion of the sensitivity of τ_D_ on the CP-MCSCF convergence threshold presented in Figure S2. As mentioned earlier, the photodynamics of 1,2-dithiane is characterized by the fact that three singlet electronic states become degenerate upon ring opening following a S–S bond dissociation^28,44^ (see Figure S3 in the SI for a time trace of this bond length following photoexcitation), necessitating an accurate description of the interaction between these states dictating the transfer of nuclear amplitude. Let us first concentrate on the S_0_ population dynamics predicted by the three multiple-spawning methods (Figure 3b). OSSAIMS (ε = 0.5) reproduces the AIMS population within its standard error for the entire dynamics, while the S_0_ population trace predicted by AIMSWISS exhibits more pronounced deviations. In particular, the AIMSWISS result starts to depart significantly from the AIMS reference at around 60 fs, reentering the standard error of the AIMS result again at ∼80 fs (with only a brief deviation later on, at ∼105 and ∼125 fs). One could expect AIMSWISS to be more accurate than the OSSAIMS, though, since the theoretical number of electronic-structure calls—a measure of the number of couplings between TBFs to be calculated at each time step (see SI for the mathematical definition of this quantity)—of AIMSWISS is larger than that of OSSAIMS (ε = 0.5) between ∼70 and ∼110 fs (see Figure 3a). To shed light on this apparent discrepancy, one can make use of a warning system implemented with AIMSWISS that is activated whenever stochastic selections are performed prematurely. A premature selection is defined as a selection that is performed after the predicted τ_D_ has passed but where the actual overlap between the parent and child TBFs at this time is still larger than 1/e of its initial value. This simple warning checks the assumption of AIMSWISS and warns the user when a selection took place while the parent/child TBF pair may still have been interacting. Figure 3c reports histograms for the number of selection processes resulting in a warning (orange histograms) or passed (blue histograms) during the dynamics of 1,2-dithiane. During this dynamics, the number of selections taking place with a warning is 4.8 times larger than that without—a trigger for caution for the results obtained with AIMSWISS. Interestingly, a correlation can be found between the deviation of the AIMSWISS S_0_ population from the AIMS reference and an appreciable number of warnings for 50 < *t* < 75 fs (Figure 3c). This correlation at early times shows that whenever a premature stochastic selection happens it may lead to inaccuracies in the description of the coupled TBF dynamics. It is important to note, however, that the warnings issued by AIMSWISS permit to immediately detect such possible shortcomings of the method and determine an estimate for the selection parameter of a subsequent OSSAIMS calculation. This estimate is obtained by considering the set of all stochastic-selection events issuing a warning and calculating the average of the overlap that is predicted by the Gaussian decay model. This value amounts to 0.59 for 1,2-dithiane, which is close to the actual value used for the OSSAIMS dynamics ε = 0.5 and determined by preliminary runs.^28^

**Figure 3:**
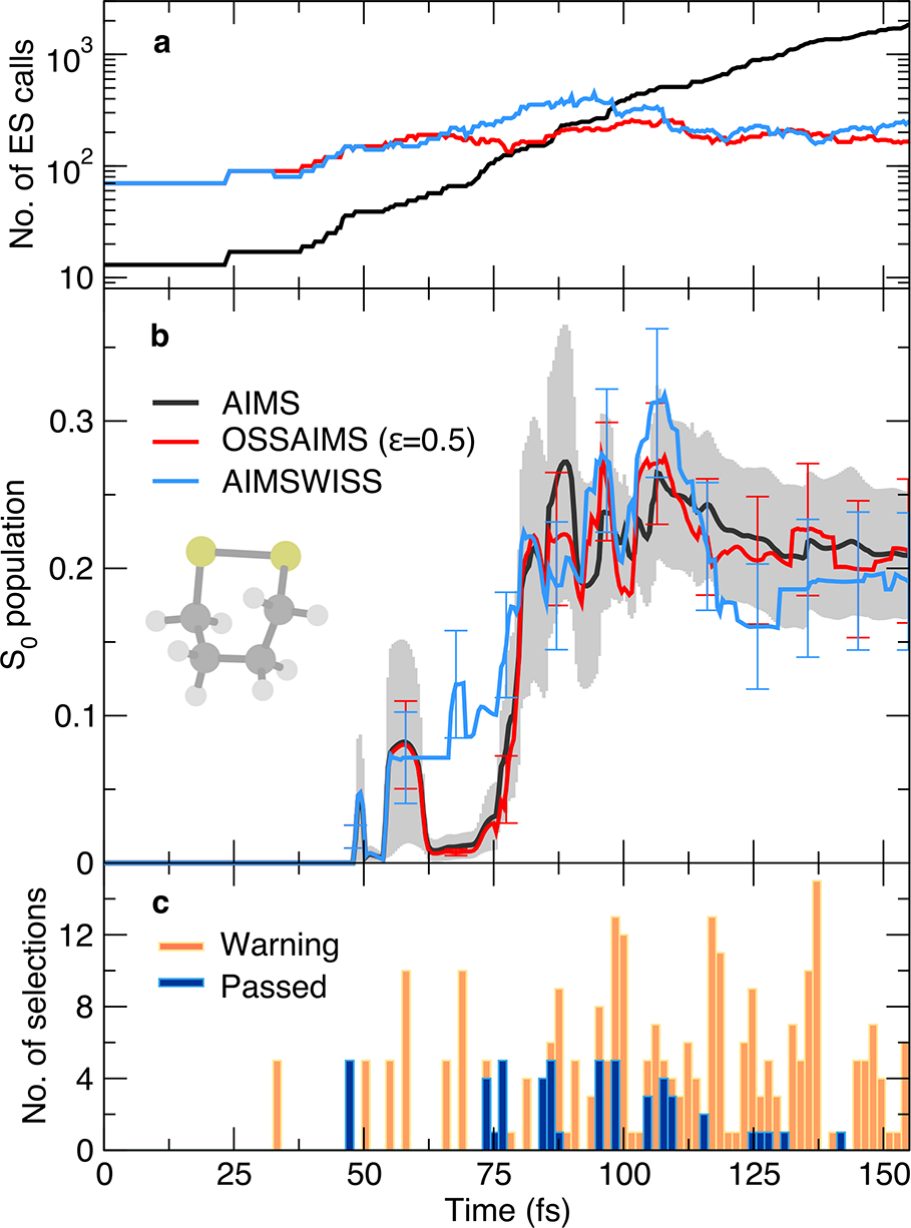
Photodynamics of 1,2-dithiane. Theoretical number of electronic-structure calls (a) and S_0_ population trace (b) for different dynamics methods: AIMS (thick black curve), OSSAIMS (red curve, ε = 0.5), and AIMSWISS (light blue curve). The standard error of the mean is indicated by a gray area for AIMS and error bars for AIMSWISS and OSSAIMS. (c) Number of selection processes in AIMSWISS resulting in a warning (orange histograms) or passed without a warning (dark blue histograms).

Let us now turn to a typical example of a photochemical process challenging the conventional multiple-spawning methods, the photodissociation of chromium (0) hexacarbonyl, [Cr(CO)_6_]. This process has been the subject of numerous experimental^45,46^ and computational investigations.^47−49^ In short, all studies agree that the UV photoexcitation of [Cr(CO)_6_] leads to formation of metal-to-ligand charge transfer (MLCT) states, triggering an excited-state dynamics that rapidly involves metal centered (MC) states causing the dissociation of a CO ligand. The large density of electronic states observed in [Cr(CO)_6_] represents a significant challenge for a multiple-spawning method as it will trigger a very fast growth of the number of TBFs. We simulated the photodynamics of this organometallic complex with AIMS, AIMSWISS, and decoherence-corrected trajectory surface hopping (dTSH), using the same set of 51 initial conditions. Five runs per initial condition were performed for AIMSWISS and dTSH, with a different seed for the random-number generator. The electronic structure of [Cr(CO)_6_] was obtained with linear-response time-dependent density functional theory (LR-TDDFT) within the Tamm-Dancoff approximation, using the B3LYP functional and considering five excited electronic states. The excited-state dynamics with AIMS and AIMSWISS was performed with FMS90, while the dTSH dynamics was conducted with the ABIN code^50^—both codes used their TeraChem interface^51,52^ for the electronic-structure calculations. Additional computational details can be found in the SI.

From a photochemical perspective, all the simulations presented here show a very similar trend for the molecular population of the singly dissociated species, [Cr(CO)_5_] (Figure 4a). The photoexcited [Cr(CO)_6_] survives for 5 fs before starting to release one of its CO ligand, and it takes 30 fs for the full population of [Cr(CO)_5_] to rise to near unity. dTSH reproduces this molecular population trace within the error bars of AIMS, whereas AIMSWISS appears to underestimate it slightly. We note that this underestimation is not due to a real deviation of AIMSWISS from AIMS but is instead caused by the way the molecular population is calculated—see Figure S4 of the SI for a discussion on this subject. We note that the time traces of the (adiabatic) electronic-state population for the three methods compared are all in close agreement (Figure S5). Monitoring the computational wall time per time step for an exemplary initial condition helps appreciate the dramatic increase in the AIMS computational cost (Figure 4c). During the first 5 fs of dynamics, AIMS and AIMSWISS take the same amount of time to complete a propagation time step—the spikes in wall time occur whenever a spawning event takes place and is due to the spawning algorithm. A divergence between the two methods start to be observed after 5 fs, where the wall time for AIMS time step increases rapidly due to the increase in the number of TBFs—leading to a large number of electronic-structure calculations to determine their couplings. Within 10 fs of dynamics, the AIMS calculation already takes more than 2 h 45 min per time step on an NVIDIA Tesla V100 GPU and one CPU thread running on a 32-core Intel Xeon Gold 6130 CPU. In contrast, AIMSWISS never takes more than 45 min per time step. With such a large wall time per time step, the AIMS simulation became prohibitively expensive already after ∼15 fs of propagation and was stopped at this point—this is the reason why all AIMS observables presented here for this molecule are shown only until this maximum time. Turning now to the overall number of electronic-structure calls (Figure 4b), dTSH—which is run five times per initial condition such that its error bars agree with those of AIMSWISS—is the most computationally efficient method, and AIMSWISS appears to be more expensive than AIMS in the early stage of the dynamics. This apparent increase of the computational cost is due to the fact that each AIMSWISS initial condition is run multiple times—five times in this molecular example. Hence, while the number of runs (and therefore overall number of electronic-structure calls) is larger for AIMSWISS than AIMS at the beginning of the dynamics, each individual AIMSWISS run is dramatically cheaper than the corresponding AIMS one due to the reduced number of TBFs per run. This difference becomes noticeable after 12 fs of dynamics when the number of electronic-structure calls of AIMSWISS crosses that of AIMS.

**Figure 4:**
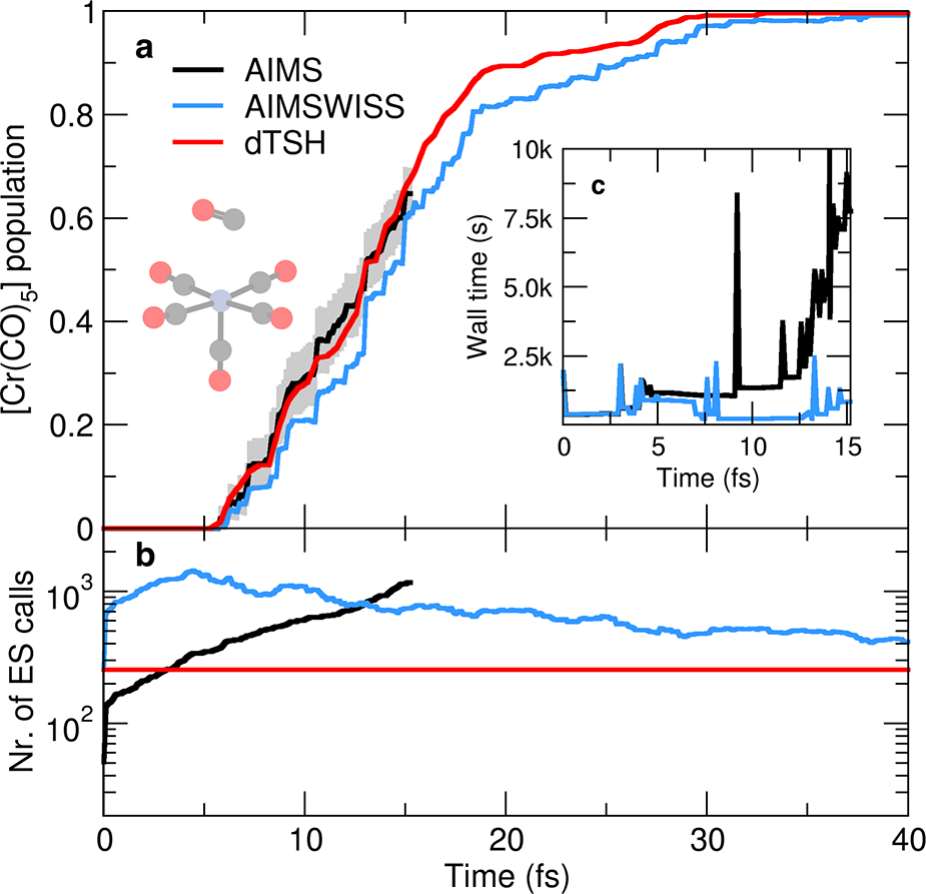
Photodissociation of chromium (0) hexacarbonyl. (a) Molecular population of chromium pentacarbonyl [Cr(CO)_5_] obtained with different nonadiabatic dynamics methods: AIMS (thick black curve), AIMSWISS (light blue curve), and dTSH (red curve). The standard error of the mean is indicated by a gray area for AIMS. (b) Theoretical number of electronic-structure calls per time step. (Inset c) Observed computational wall time per time step for the AIMS(WISS) simulation of an exemplary initial condition. Timings were recorded with one NVIDIA Tesla V100 GPU and one CPU thread running on a 32-core Intel Xeon Gold 6130 CPU.

The results obtained above show that AIMSWISS is resilient to excited-state dynamics triggering a large number of spawning events, in stark contrast with AIMS, and constitutes a viable method to validate the dynamics obtained with dTSH. The advantage of TBF-based methods over (d)TSH resides in their clear definition of a molecular wave function. Consequently, different expectation values and quantities can be directly calculated from the TBF-based molecular wave function, and we present in Figure 5a the reduced nuclear density along the Cr–C distance as obtained by AIMSWISS—a quantity formally not accessible from dTSH. Most of the reduced density is located around between 1.75 and 2 Å, while a thin contribution appears above the dividing line defining the CO dissociation after 20 fs of dynamics—spreading over time. Integrating the reduced density over a Cr–C distance larger than the dividing line gives ∼1/6, in line with the dissociation of a single CO ligand. The swarm of trajectories obtained by dTSH (Figure 5b) reproduces this trend in a purely classical picture. Finally, the reduced density offers another point of comparison between AIMSWISS and AIMS (Figure 5c), showing a nearly perfect agreement between the two methods for a snapshot at *t* ≈ 15 fs (last step of the AIMS dynamics) and further validating the accuracy of the decoupling scheme between TBFs offered by AIMSWISS. (A movie of the AIMSWISS nuclear density along the Cr–C distance is given in the SI.)

**Figure 5:**
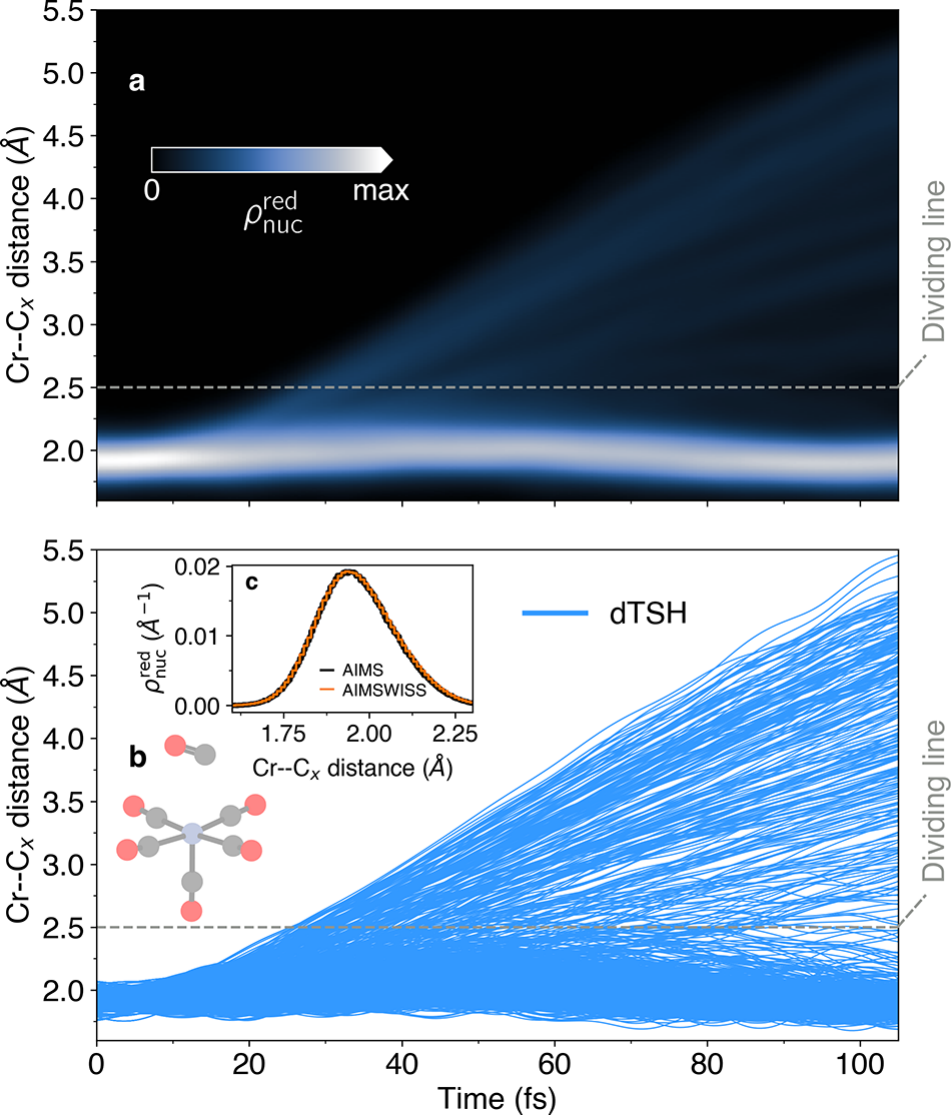
Different visualization of the CO release from chromium (0) hexacarbonyl upon photoexcitation. (a) Reduced nuclear density along the Cr–C distance obtained from the AIMSWISS dynamics. Note that the colormap corresponds to the normalized square root of the reduced nuclear density to better highlight the low-density regions. (b) Time traces of Cr–C distances resulting from the dTSH dynamics (blue curves). (Inset c) Snapshot of the reduced nuclear density along the Cr–C distance at *t* ≈ 15 fs obtained with AIMS (thick black curves) and AIMSWISS (orange curves). The dashed gray line in (a,b) represents the dividing line between [Cr(CO)_6_] and [Cr(CO)_5_].

In summary, this work dissects the performance of the AIMSWISS method for different challenging types of nonadiabatic dynamics. Using 2D model systems, we tested the resilience of the AIMSWISS algorithm to different shapes and topologies of CIs and showed that most types of CIs lead to a fast decay time and therefore efficient stochastic-selection processes. Seams of intersection may lead to an overestimation of the decay time that only impacts the efficiency of the AIMSWISS method, not its accuracy. The photodynamics of 1,2-dithiane exemplified a case where AIMSWISS can detect and report when its underlying assumptions are likely to fail, offering an opportunity to step up to the next rung of multiple-spawning methods, SSAIMS. Finally, we showed that AIMSWISS can be used in cases where a full AIMS calculation may become computational intractable—a typical example being an excited-state dynamics involving a large density of electronic states. In light of the results presented in this work, we believe that AIMSWISS can be seen as at the first rank of a hierarchy of multiple-spawning methods. We thus recommend the use of AIMSWISS as a starting method for nonadiabatic molecular dynamics simulation within the framework of multiple-spawning. For cases where AIMSWISS does not meet its own standards, the warning system in place can be used to prepare for an OSSAIMS calculation. As a last rung, AIMS dynamics can be considered, in particular when complex interactions between TBFs occur or when a specific observable requires accurate description of the coupling between TBFs. Last but not least, AIMSWISS constitutes an affordable and user-friendly strategy to benchmark mixed quantum/classical approaches for nonadiabatic molecular dynamics like TSH.

## Supporting Information Available

Computational details for all calculations, a test of the sensitivity of the AIMSWISS decay time on the convergence threshold for the nuclear gradient, a comparison between AIMS and AIMSWISS for the expectation value for the distance between the two sulfurs in 1,2-dithiane, and a sensitivity test for the molecular population of chromium (0) pentacarbonyl (PDF)
Time-evolution of the AIMSWISS reduced nuclear density along the Cr–C distance for the photodynamics of chromium (0) hexacarbonyl (MP4)
Initial conditions used for the AIMS/AIMSWISS dynamics of chromium (0) hexacarbonyl (ZIP)
Transparent Peer Review report available (PDF)
