# Supplementary material for: Extending the Applicability of the Multiple-Spawning Framework for Nonadiabatic Molecular Dynamics
Source: J Phys Chem Lett. 2022 Dec 21;13(51):12011–8. doi: 10.1021/acs.jpclett.2c03295 (PMC9806853; doi:10.1021/acs.jpclett.2c03295)
Supplement: Supplementary file 1 — jz2c03295_si_001.pdf [file jz2c03295_si_001.pdf]

Supporting Information: Extending the Applicability of the Multiple-Spawning Framework for Nonadiabatic Molecular Dynamics

Yorick Lassmann, Daniel Hollas, and Basile F. E. Curchod*

Centre for Computational Chemistry, School of Chemistry, University of Bristol, Bristol BS8 1TS, UK

E-mail: basile.curchod@bristol.ac.uk

Computational Details

Electronic Structure

The electronic structure of the molecules discussed in the main text – 1,2-dithiane and chromium (0) hexacarbonyl – was calculated with the GPU-accelerated TeraChem software package.[S1–S5] Ground state properties of chromium (0) hexacarbonyl that are used for sampling initial conditions were calculated with the Gaussian 09 software package.[S6] As done in Ref. S7, the electronic structure of 1,2-dithiane was described at the state-averaged complete active space self-consistent field (SA-CASSCF) level of theory with a three-state averaging and a (6,4) active space, comprised by one pair of $\sigma\sigma^*$ orbitals of the S−S bond and two sulfur lone pairs n_S, using the 6-31G* basis set.[S8] The electronic structure of chromium (0) hexacarbonyl was simulated at the linear-response time-dependent density functional theory (LR-TDDFT) level of theory with the B3LYP exchange-correlation functional and the Tamm-Dancoff approximation, as done in Ref. S9. We used here the LANL2DZ[S10–S12] effective core potential for Cr and the DZVP[S13] basis set for all other atoms. A validation of the basis set against the one used in Ref. S9 was performed for the photoabsorption cross-section of the molecule. For the three two-state two-dimensional model systems – BMA, butatriene cation, and pyrazine – the same parameters were used as in Refs. S14,S15 to get the diabatic and adiabatic PESs.

Nuclear Dynamics

For the dynamics on each two-state two-dimensional model system, the 2000 initial conditions (ICs) needed for AIMS and AIMSWISS were sampled from a Wigner distribution of uncoupled harmonic oscillators centered at the Franck-Condon point in configuration space with a width determined by the parameters of the model.[S15] Note that the Wigner distribution is centered at zero in momentum space for all models. The initial Gaussian wavepacket for each QD

calculations corresponds to the electronic and vibrational ground-state wavefunction projected onto the excited state. The TBFs used in the AIMS(WISS) dynamics had the same widths as the initial Gaussian wavepackets.

For all molecular systems, the ICs (14 for 1,2-dithiane and 51 for chromium (0) hexacarbonyl) were sampled from a Wigner distribution of uncoupled harmonic oscillators, employing the normal modes of the minimum-energy ground-state geometry as input. In the case of chromium (0) hexacarbonyl, the chromium width parameter was determined to be 29.49 bohr^{-2} via the nonlinear optimization method described in the Supporting Information of Ref. S16 and using the code optimwidths.[S17] For all other atoms the standard width parameters were used.[S18] The ICs of chromium (0) hexacarbonyl could be started from different excited states ($S_1 - S_3$), and the selected electronic state for each IC was that with the highest oscillator strength. For chromium (0) hexacarbonyl all ICs and their respective selected electronic state are attached to this Supplementary Information.

The AIMS, AIMSWISS, and OSSAIMS nuclear dynamics for the two molecules were performed employing FMS90 (developed by Prof Todd J. Martínez, Stanford University) interfaced with the TeraChem software package.[S19] The AIMS and AIMSWISS dynamics for the two-dimensional models were performed using FMS90 as implemented in MOLPRO,[S20] while the QD was done in the diabatic basis employing the split-operator formalism.[S21] The time step for (OSS)AIMS and AIMSWISS simulations in the case of 1,2-dithiane and chromium (0) hexacarbonyl was set to 20 atomic time units (atu), further reduced to 5 atu in regions of strong couplings. The time step was set to 1 atu for the two-dimensional models, and reduced to 0.25 atu in high-coupling regions. The effective nonadiabatic coupling threshold to enter a spawning mode was fixed to 0.0001 au^{-1} for all two-dimensional models (magnitude of the nonadiabatic coupling vectors), 20.0 au^{-1} for 1,2-dithiane (magnitude of the nonadiabatic coupling vectors), and 0.0025 au^{-1} for chromium hexacarbonyl (projection of the nonadiabatic coupling vectors onto the TBF nuclear velocity). The minimum population

for a TBF to be allowed to spawn was set to 0.001 for all two-dimensional models, 0.05 for 1,2-dithiane, and 0.1 for chromium hexacarbonyl. TBFs are only created when their overlap with the rest of the TBF basis set is below 0.6 in the case of the two-dimensional models and chromium hexacarbonyl, while this threshold is fixed to 0.5 for 1,2-dithiane. The energy difference between electronic states below which nonadiabatic couplings are calculated is set to 0.04 au for 1,2-dithiane, 0.03 au for the two-dimensional models, and 0.02 au for chromium hexacarbonyl. During the AIMS dynamics of 1,2-dithiane and the two-dimensional models (and OSSAIMS dynamics of 1,2-dithiane), TBFs are removed on the ground state if they become fully decoupled from the rest of the basis set (i.e., when their Hamiltonian matrix element drops below 10^{-10} au) for more than 200 atu. This strategy is not necessary when using AIMSWISS. In the case of chromium (0) hexacarbonyl, the dynamics was stopped as soon as a TBF moving on the first excited electronic state crosses with the ground state.

The dTSH dynamics of chromium (0) hexacarbonyl were performed with ABIN,[S22] interfaced with the TeraChem software package. Each initial condition was run five times until the error bars, estimated by the standard error of the quantum amplitudes, agreed with the ones of AIMSWISS. The time step was set to 20 atu. The maximum energy difference for which nonadiabatic couplings are calculated was set to 0.02 au. A minimum population of 0.001 was required for a given electronic state to calculate the nonadiabatic couplings with the other electronic states.

The worst case number of electronic-structure (ES) calls (see Fig. 3a and Fig. 4b of the main text) was determined via the following, equation

$$N_{\text{ES}}(t) = \frac{1}{2} \sum_{j=1}^{n_{\text{run}}} \sum_{k}^{N_{\text{IC}}} N_{\text{TBF}}^{j,k}(t) \left[N_{\text{TBF}}^{j,k}(t) + 1 \right] , \tag{1}$$

where $N_{\text{TBF}}^{j,k}(t)$ is the number of TBFs at time t for a given initial condition k (out of N_{IC} ICs) and its jth repetition or run (our of n_{run}). $n_{\text{run}} = 1$ for AIMS (or AIMSWISS with only

one repetition). The number of electronic-structure calls per time step for dTSH is simply $n_{\text{run}} N_{\text{IC}}$.

To summarize, the following values were used in this work. For the two-dimensional models, $N_{\text{IC}} = 2000$ and $n_{\text{run}} = 1$ for AIMS, and $N_{\text{IC}} = 2000$ and $n_{\text{run}} = 3$ for the converged AIMSWISS. For 1,2-dithiane, $N_{\text{IC}} = 14$ and $n_{\text{run}} = 1$ for AIMS, and $N_{\text{IC}} = 14$ and $n_{\text{run}} = 5$ for the converged AIMSWISS (and OSSAIMS). For chromium (0) hexacarbonyl, $N_{\text{IC}} = 51$ and $n_{\text{run}} = 1$ for AIMS, $N_{\text{IC}} = 51$ and $n_{\text{run}} = 5$ for the converged AIMSWISS, and $N_{\text{IC}} = 51$ and $n_{\text{run}} = 5$ for the dTSH.

The calculation of the reduced nuclear density from the AIMS and AIMSWISS were performed with the software suite pydynpost – a set of postprocessing scripts for both AIMS and TSH methods.[S23]

Analysis of the decay time τ_D for the three 2D model systems

Uncovering the origin of the difference in efficiency of the AIMSWISS selection process between the three 2D model systems necessitates an analysis of the behavior of the calculated decay time for the different CIs in presence. We calculated the model decay times τ_D for all spawning geometries generated during the AIMS dynamics and compared them to the decay times τ_{AIMS} actually observed in AIMS. τ_{AIMS} is the time required for the overlap of a parent-child TBF pair to decay to reach 1/e of its initial value. The spawning geometries were then grouped into two categories: spawning geometries for which τ_D overestimates τ_{AIMS} by more than a factor two, and spawning geometries where it does not. We then calculated τ_D for all possible nuclear configurations covered by the (analytical) two-dimensional models – creating a heat map of the decay time for any point of configuration space – and superimposed the spawning geometries on this heat map. Figure S1a-c provide the resulting plots for the three models considered, with the heat map representing the decay time and the spawning geome-

tries with $\tau_D \geq 2\tau_{AIMS}$ symbolized by gray circles while those with $\tau_D < 2\tau_{AIMS}$ are indicated with cyan squares. Armed with this information we now can tackle the question as to why

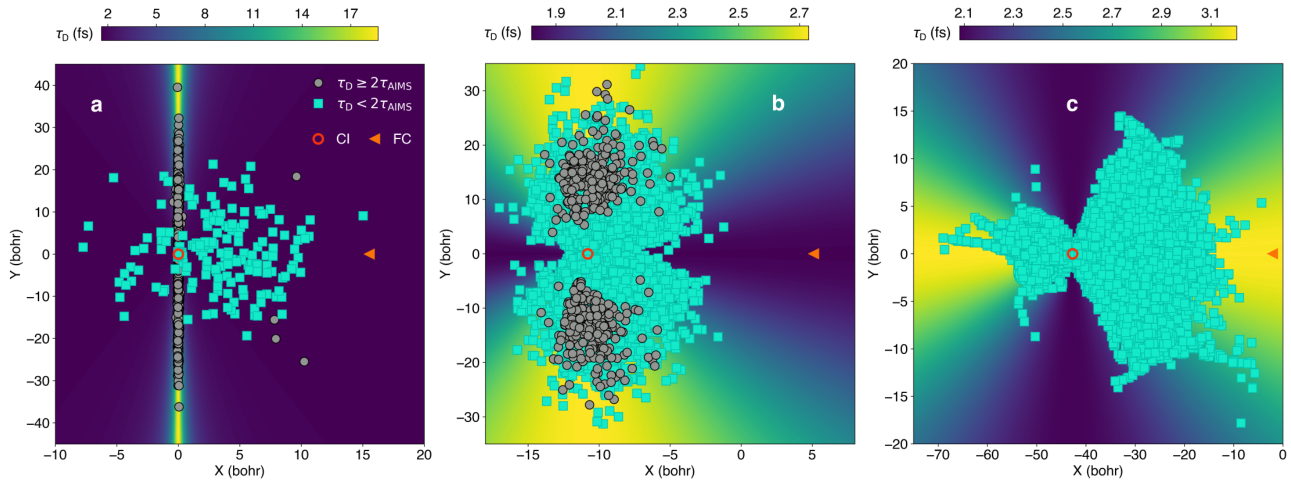

Figure S1: Spawning geometries created during the AIMS dynamics of the three two-state two dimensional model systems: (a) BMA cation, (b) butatriene cation, and (c) pyrazine. The spawning geometries are grouped in two classes – $\tau_D \geq 2\tau_{AIMS}$ (gray circles) and $\tau_D < 2\tau_{AIMS}$ (cyan squares) – and superimposed onto a heat map of the model decay time τ_D. A red circle indicates the location of the conical intersection and an orange-filled triangle that of the Franck–Condon point.

the selection dynamics of BMA differs so much from that of the other models. The CI of BMA appears peaked, but it is actually a rather special case where one of the coordinate of the branching space – the derivative coupling, Y in Fig. S1 – lifts the degeneracy between the two coupled electronic states only very weakly in comparison to the other coordinate. This ineffective removal of degeneracy along the derivative coupling coordinates means that the CI resembles more a seam of intersection. This special topology – finding its origin in a very weak diabatic coupling between the two diabatic states and, as such, a nearly vanishing derivative coupling – gives rise to the well known diabatic trapping mechanism[S24] (also known as 'up-funneling'[S25,S26]), where a molecule remains trapped within the same electronic character by efficient nonadabatic transitions and cannot evolve adiabatically on a given (adiabatic electronic state) near the seam of intersection. Such efficient nonadiabatic transitions near the seam of intersection can be observed for the BMA model, where almost perfect

population transfers occurs between the two coupled adiabatic electronic states (Fig. 2d of the main text). For a recent example of this effect in action for a molecular system, see Ref. S27. How does this seam of intersection interfere with the efficiency of AIMSWISS? In AIMSWISS, the child TBF is spawned exactly on the seam of intersection (see circles Fig. S1a), and the topography of the seam of intersection at this position means that the nuclear forces for the child and parent TBFs are similar, leading to a larger estimation of the value of τ_{D} with values up to ~ 17 fs, as clearly shown by the heat map in Fig. S1a. For comparison, other spawning events that are no more located exactly on the seam of intersection (squares in Fig. S1a) show τ_{D} values around 2 fs. Hence, as most spawning events taking place to describe the first nonadiabatic transitions occur on the seam of intersection (95 % of the total of spawning events for the BMA simulation presented here), the decay time for the AIMSWISS algorithm vastly overestimates τ_{AIMS} leading to a long time before the selection process takes place. The average number of TBFs per time step for AIMSWISS only starts to be dramatically smaller than that of AIMS after 15 fs, in agreement with the overall decay time predicted for the intersection seam. In contrast, τ_{D} varies only slightly in nuclear configuration space for the more conventional conical intersections observed for the butatriene cation and pyrazine models (see the range of the heat map in Fig. S1b-c). This rather homogeneous distribution of τ_{D} explains why the amount of overestimations of τ_{AIMS} by τ_{D} is negligible for these models – being ~ 2 % for the butatriene cation and 0 % for pyrazine.

Supplementary figures

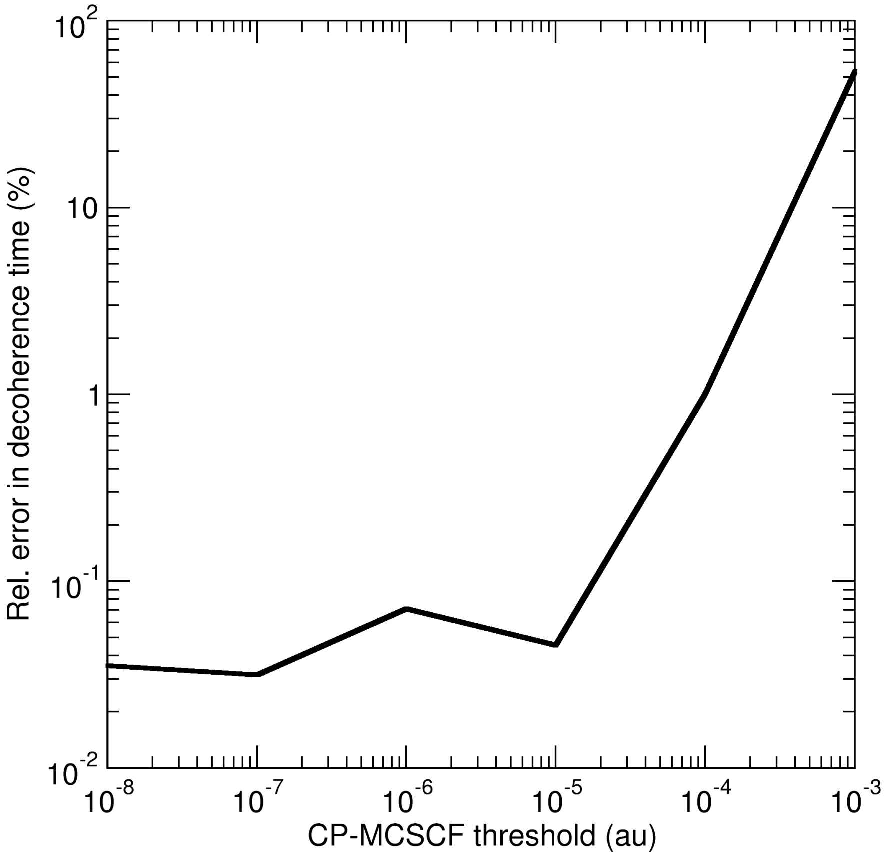

Figure S2: Dependency of the decoherence time on the convergence threshold of the coupled-perturbed multiconfigurational self consistent field (CP-MCSCF) algorithm for calculating the nuclear gradients. The results presented here were calculated from a single AIMSWISS run of 1,2 dithiane, starting from the same IC for each CP-MCSCF threshold. Decreasing this convergence threshold to under 10^{-4} au leads to a variation in decoherence time smaller than 1%.

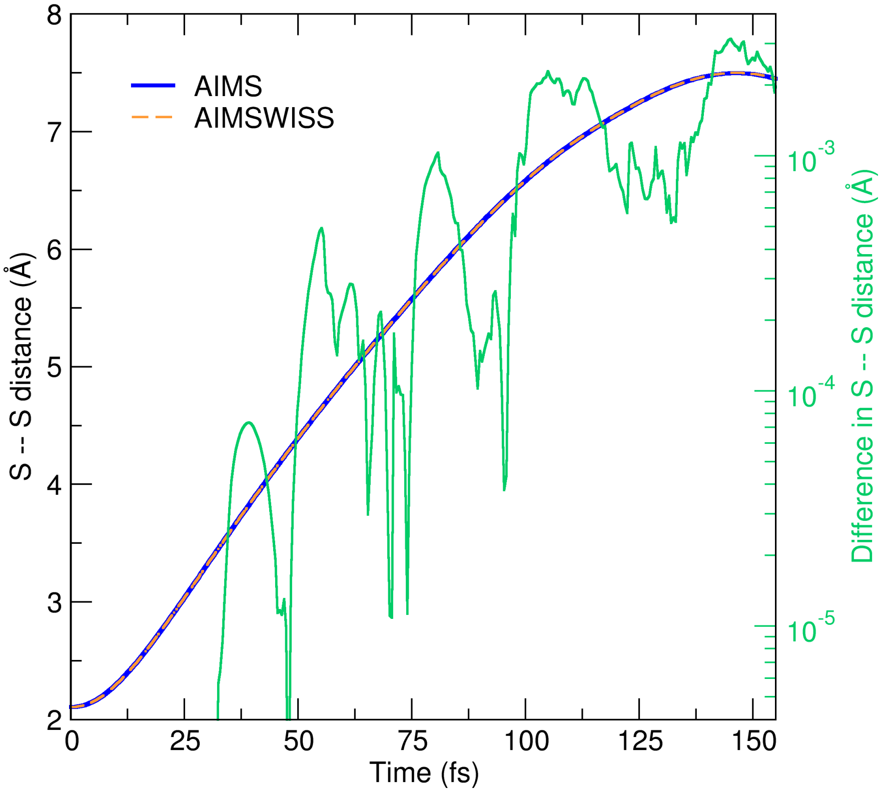

Figure S3: Time trace of the expectation value of the disulfide bond length (S–S distance) during the photodynamics of 1,2-dithiane (calculated via an incoherent sum over TBFs). The S–S distance dynamics of AIMS (blue curve) is indistinguishable from that of AIMSWISS (orange dashed curve). The deviation between the two time traces is shown with a green curve.

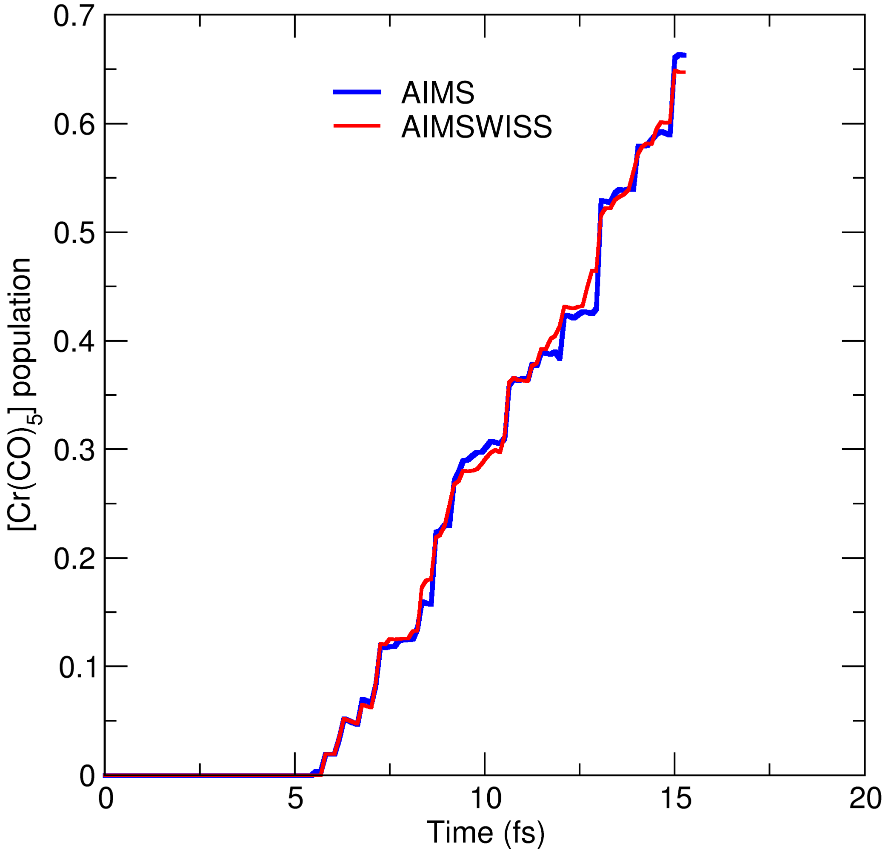

Figure S4: Time trace of the molecular population of chromium (0) pentacarbonyl, obtained from AIMS (blue curve) and AIMSWISS (red curve). The difference between the AIMSWISS molecular population here and the one depicted in Fig. 4 in the main text is that the end point for the analysis of the nuclear dynamics determines which bond will be considered dissociated. In the calculation of the molecular population, only the bond with the largest Cr—C distance above the dissociation threshold is considered dissociated. However, because a Cr—C bond, previously presumed dissociated, can recross the dissociation threshold and thus be reformed, the calculation of the molecular population is sensitive to the dissociation threshold and the geometry at the last time step that is used to determine the dissociated bond.

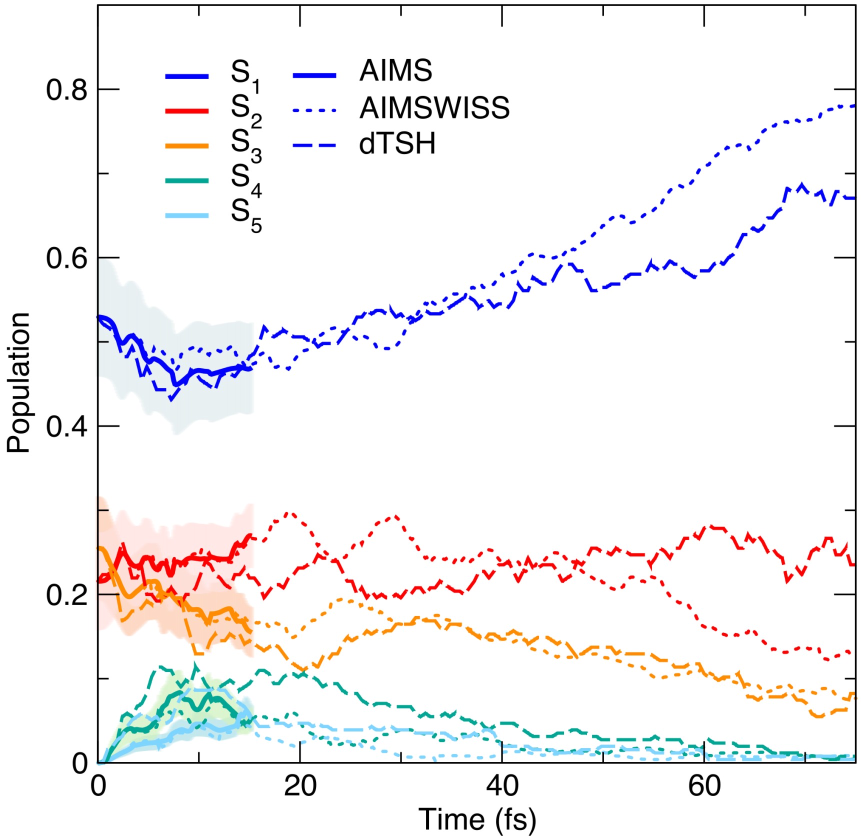

Figure S5: Time traces of the adiabatic state populations for all five electronic states involved in the dynamics of chromium (0) hexacarbonyl, calculated with AIMS (thick curves), AIMSWISS (dotted curves), and dTSH (dashed curves). The shaded areas correspond to the standard error of the mean for AIMS. The three methods agree well in their resulting adiabatic state populations within the first 15 fs of dynamics, while a small deviation between dTSH and AIMSWISS becomes visible at later time, especially for the S_1, S_2, and S_4 states. At the end of the dynamics (at around 75 fs), the S_1 population predicted by dTSH deviates by more than 10 % from that of AIMSWISS.

References

(S1) Ufimtsev, I. S.; Martínez, T. J. Quantum Chemistry on Graphical Processing Units. 1. Strategies for Two-Electron Integral Evaluation. *J. Chem. Theory Comput.* **2008**, *4*, 222–231.

(S2) Ufimtsev, I. S.; Martínez, T. J. Quantum Chemistry on Graphical Processing Units.

2. Direct Self-Consistent-Field Implementation. *J. Chem. Theory Comput.* **2009**, *5*, 1004–1015.

(S3) Ufimtsev, I. S.; Martínez, T. J. Quantum Chemistry on Graphical Processing Units. 3. Analytical Energy Gradients, Geometry Optimization, and First Principles Molecular Dynamics. *J. Chem. Theory Comput.* **2009**, *5*, 2619–2628.

(S4) Seritan, S.; Bannwarth, C.; Fales, B. S.; Hohenstein, E. G.; Kokkila-Schumacher, S. I. L.; Luehr, N.; Snyder, J. W.; Song, C.; Titov, A. V.; Ufimtsev, I. S.; Martínez, T. J. TeraChem: Accelerating Electronic Structure and *Ab Initio* Molecular Dynamics with Graphical Processing Units. *J. Chem. Phys.* **2020**, *152*, 224110.

(S5) Seritan, S.; Bannwarth, C.; Fales, B. S.; Hohenstein, E. G.; Isborn, C. M.; Kokkila-Schumacher, S. I. L.; Li, X.; Liu, F.; Luehr, N.; Snyder, J. W.; Song, C.; Titov, A. V.; Ufimtsev, I. S.; Wang, L.-P.; Martínez, T. J. TeraChem: A Graphical Processing Unit - Accelerated Electronic Structure Package for Large-scale Ab Initio Molecular Dynamics. *WIREs Comput. Mol. Sci.* **2020**, *11*, e1494.

(S6) Frisch, M. J.; Trucks, G. W.; Schlegel, H. B.; Scuseria, G. E.; Robb, M. A.; Cheeseman, J. R.; Scalmani, G.; Barone, V.; Mennucci, B.; Petersson, G. A.; Nakatsuji, H.; Caricato, M.; Li, X.; Hratchian, H. P.; Izmaylov, A. F.; Bloino, J.; Zheng, G.; Sonnenberg, J. L.; Hada, M.; Ehara, M.; Toyota, K.; Fukuda, R.; Hasegawa, J.; Ishida, M.; Nakajima, T.; Honda, Y.; Kitao, O.; Nakai, H.; Vreven, T.; J. A., M., Jr.; J. E. Peralta,; F. Ogliaro,; M. Bearpark,; J. J. Heyd,; E. Brothers,; K. N. Kudin,; V. N. Staroverov,; T. Keith,; R. Kobayashi,; J. Normand,; K. Raghavachari,; A. Rendell,; J. C. Burant,; S. S. Iyengar,; J. Tomasi,; M. Cossi,; N. Rega,; J. M. Millam,; M. Klene,; J. E. Knox,; J. B. Cross,; V. Bakken,; C. Adamo,; J. Jaramillo,; R. Gomperts,; R. E. Stratmann,; O. Yazyev,; A. J. Austin,; R. Cammi,; C. Pomelli,; J. W. Ochterski,; R. L. Martin,; K. Morokuma,; V. G. Zakrzewski,; G. A. Voth,; P. Salvador,; J. J.

Dannenberg,; S. Dapprich,; A. D. Daniels,; O. Farkas,; J. B. Foresman,; J. V. Ortiz,; J. Cioslowski,; D. J. Fox, Gaussian 09, Revision D.01. Gaussian, Inc., 2013.

(S7) Ibele, L. M.; Lassmann, Y.; Martínez, T. J.; Curchod, B. F. E. Comparing (Stochastic-Selection) *Ab Initio* Multiple Spawning with Trajectory Surface Hopping for the Photodynamics of Cyclopropanone, Fulvene, and Dithiane. *J. Chem. Phys.* **2021**, *154*, 104110.

(S8) Ditchfield, R.; Hehre, W. J.; Pople, J. A. Self-Consistent Molecular-Orbital Methods. IX. An Extended Gaussian-Type Basis for Molecular-Orbital Studies of Organic Molecules. *The Journal of Chemical Physics* **1971**, *54*, 724–728.

(S9) Crespo-Otero, R.; Barbatti, M. $Cr(CO)_6$ Photochemistry: Semi-classical Study of UV Absorption Spectral Intensities and Dynamics of Photodissociation. *J. Chem. Phys.* **2011**, *134*, 164305.

(S10) Hay, P. J.; Wadt, W. R. *Ab Initio* Effective Core Potentials for Molecular Calculations. Potentials for the Transition Metal Atoms Sc to Hg. *J. Chem. Phys.* **1985**, *82*, 270–283.

(S11) Wadt, W. R.; Hay, P. J. *Ab Initio* Effective Core Potentials for Molecular Calculations. Potentials for Main Group Elements Na to Bi. *J. Chem. Phys.* **1985**, *82*, 284–298.

(S12) Hay, P. J.; Wadt, W. R. *Ab Initio* Effective Core Potentials for Molecular Calculations. Potentials for K to Au Including the Outermost Core Orbitals. *J. Chem. Phys.* **1985**, *82*, 299–310.

(S13) Chiodo, S.; Russo, N.; Sicilia, E. Newly Developed Basis Sets for Density Functional Calculations. *J. Comput. Chem.* **2005**, *26*, 175–184.

(S14) Ryabinkin, I. G.; Joubert-Doriol, L.; Izmaylov, A. F. When Do We Need to Account for the Geometric Phase in Excited State Dynamics? *J. Chem. Phys.* **2014**, *140*, 214116.

(S15) Ibele, L. M.; Curchod, B. F. E. Dynamics near a Conical Intersection—A Diabolical Compromise for the Approximations of *Ab Initio* Multiple Spawning. *J. Chem. Phys.* **2021**, *155*, 174119.

(S16) Esch, M. P.; Shu, Y.; Levine, B. G. A Conical Intersection Perspective on the Low Nonradiative Recombination Rate in Lead Halide Perovskites. *J. Phys. Chem. A* **2019**, *123*, 2661–2673.

(S17) Lassmann, Y. optimwidths: Tool for calculating the Gaussian width parameters of AIMS. Last accessed: November 2022. 2022; `https://doi.org/10.5281/zenodo.7382685`.

(S18) Thompson, A. L.; Punwong, C.; Martínez, T. J. Optimization of Width Parameters for Quantum Dynamics with Frozen Gaussian Basis Sets. *Chem. Phys.* **2010**, *370*, 70–77.

(S19) Curchod, B. F. E.; Sisto, A.; Martínez, T. J. Ab Initio Multiple Spawning Photochemical Dynamics of DMABN Using GPUs. *J. Phys. Chem. A* **2017**, *121*, 265–276.

(S20) Levine, B. G.; Coe, J. D.; Virshup, A. M.; Martínez, T. J. Implementation of Ab Initio Multiple Spawning in the Molpro Quantum Chemistry Package. *Chem. Phys.* **2008**, *347*, 3–16.

(S21) Feit, M. D.; Fleck, J. A.; Steiger, A. Solution of the Schrödinger Equation by a Spectral Method. *J. Comput. Phys* **1982**, *47*, 412–433.

(S22) Hollas, D.; Suchan, J.; Svoboda, O.; Ončák, M.; Slavíček, P. ABIN: Multipurpose ab initio MD program. Last accessed: November 2022. 2022; `https://doi.org/10.5281/zenodo.1228462`.

(S23) Lassmann, Y. pydynpost: Postprocessing tools for nonadiabatic molecular dynamics methods. Last accessed: November 2022. 2022; `https://doi.org/10.5281/zenodo.7382636`.

(S24) Blancafort, L.; Hunt, P.; Robb, M. A. Intramolecular Electron Transfer in Bis(Methylene) Adamantyl Radical Cation: A Case Study of Diabatic Trapping. *J. Am. Chem. Soc.* **2005**, *127*, 3391–3399.

(S25) Martínez, T. J. Ab Initio Molecular Dynamics around a Conical Intersection: Li(2p) + H_2. *Chem. Phys. Lett.* **1997**, *272*, 139–147.

(S26) Ko, C.; Levine, B.; Toniolo, A.; Manohar, L.; Olsen, S.; Werner, H.-J.; Martínez, T. J. Ab Initio Excited-State Dynamics of the Photoactive Yellow Protein Chromophore. *J. Am. Chem. Soc.* **2003**, *125*, 12710–12711.

(S27) Marsili, E.; Prlj, A.; Curchod, B. F. E. A Theoretical Perspective on the Actinic Photochemistry of 2-Hydroperoxypropanal. *J. Phys. Chem. A* **2022**, *126*, 5420–5433.
