# Supplementary material for: Extending the Applicability of the Multiple-Spawning Framework for Nonadiabatic Molecular Dynamics
Source: J Phys Chem Lett. 2022 Dec 21;13(51):12011–8. doi: 10.1021/acs.jpclett.2c03295 (PMC9806853; doi:10.1021/acs.jpclett.2c03295)
Supplement: Supplementary file 4 — jz2c03295_si_004.pdf [file jz2c03295_si_004.pdf]

jz-2022-032959.R1

Name: Peer Review Information for "Extending the Applicability of the Multiple-Spawning Framework for Nonadiabatic Molecular Dynamics"

First Round of Reviewer Comments

Reviewer: 1

Comments to the Author

The paper dealing with the AIM techniques and its variations, restrictions, and approximations is well written and clearly presented. These theories have been generated previously but in this paper the authors apply the basic ideas, which they have helped develop, to a few molecular systems. The work is interesting and will be useful got the community of physical chemists interested in calculations of reaction dynamics in complex molecular species.

My only concern about publication in JPClett is that the work is not really of immediate and important concern to the interested audience. I might think that the paper could easily appear in JPCA or even JCP and might have a wider impact. I think the final judgement of this suggestion might be left to the Editor and the authors.

Notwithstanding, the paper should be published as submitted.

Reviewer: 2

Comments to the Author

This work presents a rigorous computational study on the performance of the AIMSWISS (Ab initio multiple spawning with informed stochastic selections) approach recently proposed by Y. Lassmann and B. F. E. Curchod, *J. Chem. Phys*. **154**, 211106 (2021). The authors show this parameter-free strategy leads to a near quantitative agreement with AIMS populations in representative and challenging photodynamics model systems and molecules, with a significantly reduced computational time. This work shows AIMSWISS to be a robust and computationally efficient method -with running times comparable to TSH-, opening the possibility of performing viable and systematic calculations on large systems while keeping the accuracy of AIMS (Ab Initio Multiple Spawning methods). The concordance between the reduced nuclear density along the molecular bond length -a quantity that can not be described by independent trajectory methods such as TSH or Ehrenfest-, represents additional proof of the reliability of the AIMSWISS approach.

The manuscript is correctly prepared, carefully and clearly written and the presented content is understandable for the reader. I highly appreciate the work put into performing the research and presenting the results. Considering the notorious difficulty of consistently improving the applicability of multiple spawning methods to large molecular systems while retaining their accuracy and reliability, the present work represents a significant step forward and I highly recommend its publication in JPCL.

Yet, I have some questions/suggestions, that I kindly ask the authors to address.

1. Figure 1 shows a schematic representation of the AIMSWISS approach. Although it is very helpful to follow the description in the text, I would appreciate if the authors could clarify some details. In the manuscript, the authors describe: "…at some point spawns a child, which is included into the basis set with zero population and back-propagated to the time at which the parent TBF entered spawning mode…" (Pg. 5, 46-50), which refers to step (i). Is this spawning point corresponding to step (ii)? Does (i) represent the evolution of the initial TBF from t=0 to $t=t_{spawn}$? Maybe, t_{spawn} can be also labeled in the figure.

   I assume, from the figure, that the thick green line corresponds to the parent-child TBFs pair overlap, I suggest including this information in the figure caption.

2. On pg.6, 38-42, "…two groups of TBFs – the one comprised of the child TBF and its progeny, and the other one containing the parent TBF and its other offspring – will become disconnected after the time t_D has elapsed and one of the groups will be selected…" How does this selection ensure you are not missing crucial information from the discarded group of TBFs?

3. Figure 3, shows a grey shadow along the AIMS population trace. I assume it is the AIMS corresponding standard error, but I suggest the authors to add that information in the manuscript.

4. For BMA model, the authors show the predicted t_D overestimates the actual AIMS overlap decay, leading to the lower efficiency of AIMSWISS in preventing the growth of the number of TBFs. This effect is attributed to the shape and topology of the CI. Suppose one wants to use AIMSWISS as a predictive method of the dynamics of a given molecule without knowing the topology of the PES. How could one avoid falling into large computational times comparable to AIMS? Is there any possible systematic way to know if AIMSWISS will suffer from the t_D overestimation problem?

5. Figure 3 shows the ground state population traces for three different multiple spawning methods (AIMS, OSSAIMS and AIMSWISS). The authors conclude that there is a correlation between the failure of AIMSWISS to predict the correct dynamics, compared to AIMS and OSSAIMS, and the number of premature selections done by AIMSWISS. From the plot, panels b and c, the number of selections resulting in a warning between ~60-75 fs is similar to those at ~50-60 fs, and smaller than the "warning" selections at ~100 fs, but the deviation of the AIMSWISS population trace from AIMS or OSSAIMS is larger during the 60-75 fs time interval. Could the authors explain this observation?

6. AIMSWISS method significantly reduces the computational time compared to AIMS, but still the authors show it is not as affordable as TSH methods. In a previous work (*J. Chem. Phys*. **154**, 104110 (2021)), OSSAIMS is found to reproduce AIMS results at similar computational cost than TSH. Can the authors provide an estimate on computational cost of AIMSWISS compared to TSH, in situations -like butatriene cation or pyridine models- where the algorithm efficiently prevents the growth of number of TBFs at short times?

7. Why is the number of IC chosen so low for 1,2 dithiane (18) and chromium (0) hexacarbonil (51)? Is this due to electronic structure instabilities in the AIMS dynamics when including other ICs obtained from a Wigner distribution? Does AIMSWISS suffer from the same problem? Can AIMSWISS converge faster including more ICs and fewer runs?

8. Figure S5 shows the AIMSWISS population traces follow quite closely those predicted by AIMS, while dTSH starts to deviate at early times, but still falls within the AIMS standard error. AIMSWISS offers accuracy and the possibility of measuring quantities unaccessible to TSH, but for large/challenging systems, where and how would someone establish the compromise between accuracy and speed, that is, when AIMSWISS would be a preferable method over decoherence corrected independent trajectory approaches (TSH, Ehrenfest) to simulate the dynamics of a photoexcited system?

Author's Response to Peer Review Comments:

Basile F. E. Curchod
Associate Professor
Centre for Computational Chemistry
School of Chemistry, University of Bristol
Bristol BS8 1TS
United Kingdom
E: basile.curchod@bristol.ac.uk
T: (+44) 757 704 04 24
W: www.in-silico-photochem.com

December 1, 2022

Prof. Editor
Senior Editor
The Journal of Physical Chemistry Letters

Dear Prof. Editor,

We thank the two Reviewers for their very positive and constructive comments on our manuscript "Extending the Applicability of the Multiple-Spawning Framework for Nonadiabatic Molecular Dynamics" (ID: jz-2022-032959). We have updated the manuscript to address all the Reviewers' suggestions. We detail these changes and include responses to the Reviewers' questions below. We reproduce below the entire Reviewer comments in red for clarity, and additions/corrections to the text are denoted in blue. We also uploaded an annotated version of the revised manuscript, where all modifications are indicated in blue.

Report of Reviewer #1

The paper dealing with the AIM techniques and its variations, restrictions, and approximations is well written and clearly presented. These theories have been generated previously but in this paper the authors apply the basic ideas, which they have helped develop, to a few molecular systems. The work is interesting and will be useful got the community of physical chemists interested in calculations of reaction dynamics in complex molecular species.

My only concern about publication in JPClett is that the work is not really of immediate and important concern to the interested audience. I might think that the paper could easily appear in JPCA or even JCP and might have a wider impact. I think the final judgement of this suggestion might be left to the Editor and the authors.

Notwithstanding, the paper should be published as submitted.

We thank the Reviewer for their positive comments on our manuscript. The Reviewer does not request any modification on our manuscript and suggests to publish it *as submitted*. The only comment made by Reviewer #1 is about the immediacy and importance of the work and if the Journal of Physical Chemistry Letters is the best place to publish it – leaving it to the Editor and the authors to decide. We strongly believe that the Journal of Physical Chemistry Letters is the best venue for our article as numerous important developments in nonadiabatic dynamics have been published in its pages and this Journal is closely followed by the community of computational photochemists. More importantly, we stress that Reviewer #2 noted the significant step forward that our work represent for the multiple spawning methods and *highly recommend* its publication in the Journal of Physical Chemistry Letters.

Report of Reviewer #2

This work presents a rigorous computational study on the performance of the AIMSWISS (Ab initio multiple spawning with informed stochastic selections) approach recently proposed by

Y. Lassmann and B. F. E. Curchod, J. Chem. Phys. 154, 211106 (2021). The authors show this parameter-free strategy leads to a near quantitative agreement with AIMS populations in representative and challenging photodynamics model systems and molecules, with a significantly reduced computational time. This work shows AIMSWISS to be a robust and computationally efficient method -with running times comparable to TSH-, opening the possibility of performing viable and systematic calculations on large systems while keeping the accuracy of AIMS (Ab Initio Multiple Spawning methods). The concordance between the reduced nuclear density along the molecular bond length -a quantity that can not be described by independent trajectory methods such as TSH or Ehrenfest-, represents additional proof of the reliability of the AIMSWISS approach.

The manuscript is correctly prepared, carefully and clearly written and the presented content is understandable for the reader. I highly appreciate the work put into performing the research and presenting the results. Considering the notorious difficulty of consistently improving the applicability of multiple spawning methods to large molecular systems while retaining their accuracy and reliability, the present work represents a significant step forward and I highly recommend its publication in JPCL.

Yet, I have some questions/suggestions, that I kindly ask the authors to address.
We thank the Reviewer for their positive and constructive comments on our manuscript.

1. Figure 1 shows a schematic representation of the AIMSWISS approach. Although it is very helpful to follow the description in the text, I would appreciate if the authors could clarify some details. In the manuscript, the authors describe: "…at some point spawns a child, which is included into the basis set with zero population and back-propagated to the time at which the parent TBF entered spawning mode…" (Pg. 5, 46-50), which refers to step (i). Is this spawning point corresponding to step (ii)? Does (i) represent the evolution of the initial TBF from t=0 to t=tspawn? Maybe, tspawn can be also labeled in the figure.

   In this part, we were using the vocabulary associated to the spawning algorithm in AIMS without identifying it. We have now clarified this in the text and could simplify the scheme presented in Figure 1.

   Page 6:

   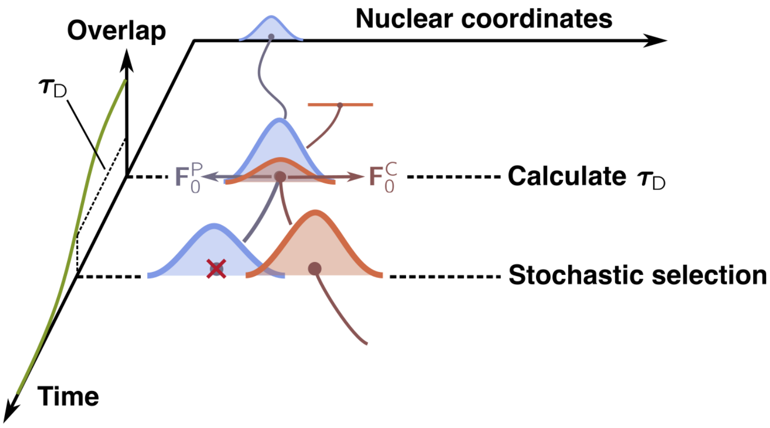

   Page 5: *An initial TBF evolves on its assigned adiabatic electronic state and can detect when it approaches a region of nonadiabaticity, triggering a 'spawning mode'. In this spawning mode, the coupled propagation of the TBFs is suspended and the parent TBF will locate the position of a maximum of the nonadiabatic coupling with another electronic state along its trajectory. The parent TBF will spawn a child TBF with zero amplitude on this coupled electronic state at the location of the maximum of the nonadiabatic coupling. The child TBF is back-propagated until the time when the parent TBF entered the spawning mode (for a detailed discussion of the spawning algorithm, please refer to Ref. 20).*

   I assume, from the figure, that the thick green line corresponds to the parent-child TBFs pair overlap, I suggest including this information in the figure caption.
   We thank the Reviewer for this suggestion and have modified the figure caption accordingly.

2. On pg.6, 38-42, "…two groups of TBFs – the one comprised of the child TBF and its progeny, and the other one containing the parent TBF and its other offspring – will become

disconnected after the time tD has elapsed and one of the groups will be selected..." How does this selection ensure you are not missing crucial information from the discarded group of TBFs?

The information is not lost as, for each initial condition, an AIMSWISS run is repeated multiple times with a different seed for the random number generator. We have modified the main text to stress this point:

> Page 7: To converge the Monte-Carlo algorithm, *and consequently to ensure that no valuable information is lost through the removal of TBFs via the stochastic selection,* AIMSWISS runs starting ...

3. Figure 3, shows a grey shadow along the AIMS population trace. I assume it is the AIMS corresponding standard error, but I suggest the authors to add that information in the manuscript.

   We thank the Reviewer for their suggestion and have changed the main text in the following way:

   > Caption Figure 3: *The standard error of the mean is indicated by a grey area for AIMS and error bars for AIMSWISS and OSSAIMS.*

   > Caption Figure 4: *The standard error of the mean is indicated by a grey area for AIMS.*

4. For BMA model, the authors show the predicted tD overestimates the actual AIMS overlap decay, leading to the lower efficiency of AIMSWISS in preventing the growth of the number of TBFs. This effect is attributed to the shape and topology of the CI. Suppose one wants to use AIMSWISS as a predictive method of the dynamics of a given molecule without knowing the topology of the PES. How could one avoid falling into large computational times comparable to AIMS? Is there any possible systematic way to know if AIMSWISS will suffer from the tD overestimation problem?

   A conceivable approach to avoid large overestimations of the decay time would be to monitor the output generated by AIMSWISS at the stochastic selection events and detect if there are many of such events where the predicted overlap is much larger than the actual one. This would be a sign that the AIMSWISS algorithm would overestimate the decay time.

5. Figure 3 shows the ground state population traces for three different multiple spawning methods (AIMS, OSSAIMS and AIMSWISS). The authors conclude that there is a correlation between the failure of AIMSWISS to predict the correct dynamics, compared to AIMS and OSSAIMS, and the number of premature selections done by AIMSWISS. From the plot, panels b and c, the number of selections resulting in a warning between ~60-75 fs is similar to those at ~50-60 fs, and smaller than the "warning" selections at ~100 fs, but the deviation of the AIMSWISS population trace from AIMS or OSSAIMS is larger during the 60-75 fs time interval. Could the authors explain this observation?

   We clarified in the main text were we observe this correlation.

   > p.12: *Interestingly, a correlation can be found between the deviation of the AIMSWISS S_0 population from the AIMS reference and an appreciable number of warnings for $50 < t < 75$ fs (Fig. 3c).*

6. AIMSWISS method significantly reduces the computational time compared to AIMS, but still the authors show it is not as affordable as TSH methods. In a previous work (J. Chem. Phys. 154, 104110 (2021)), OSSAIMS is found to reproduce AIMS results at similar computational cost than TSH. Can the authors provide an estimate on computational cost of AIMSWISS compared to TSH, in situations -like butatriene cation or pyridine models- where the algorithm efficiently prevents the growth of number of TBFs at short times?

   The comparison between the cost of AIMSWISS and TSH discussed by the Reviewer was actually performed indirectly in our original implementation of AIMSWISS (doi.org/10.1063/5.0052118), where we calculated the photodynamics of fulvene and could show that AIMSWISS was cheaper than OSSAIMS, which in turn was as expensive as TSH from the reference cited by the Reviewer. We note, however, that this applies for well converged AIMSWISS and TSH simulations – where convergence here is related to number of repetitions per initial condition for AIMSWISS and TSH, that is the convergence with respect to the number of branches for AIMSWISS and with respect to the stochastic algorithm for the nonadiabatic transitions for TSH. The purpose of our work is really not to claim that AIMSWISS 'beats' TSH in terms of computational cost, but to show that AIMSWISS is a method that retains the quality of AIMS for the description of nonadiabatic processes while dramatically cutting its computational cost, now approaching that of TSH.

7. Why is the number of IC chosen so low for 1,2 dithiane (18) and chromium (0) hexacarbonil

(51)? Is this due to electronic structure instabilities in the AIMS dynamics when including other ICs obtained from a Wigner distribution? Does AIMSWISS suffer from the same problem? Can AIMSWISS converge faster including more ICs and fewer runs?
The number of initial conditions was chosen based on the computational cost of these calculations. For 1,2-dithiane, we show the exponential cost of AIMS caused by the electronic states becoming nearly degenerate (see panel a of Fig. 3). While this number of initial conditions may look small from a TSH perspective, the number of TBFs spawned with AIMS means that this number of initial conditions already represents adequately the nonadiabatic transitions for this molecule. The same is true for the chromium (0) hexacarbonyl system (where we note that we repeated each TSH run 5 times, so in total we have 255 TSH trajectories). There were no instabilities observed during the dynamics. In AIMSWISS, the sampling of initial conditions *versus* the sampling of runs do not have the same result: the former is required for a good description of the initial state of the dynamics while the latter is required for an adequate description of the branches of TBFs obtained during the dynamics. Given that the nonadiabatic dynamics is straightforward (single crossings), meaning that the number of branches from a parent TBF will be limited, the number of AIMSWISS runs can be reduced to reproduce the AIMS result (as shown with the two-dimensional examples).

8. Figure S5 shows the AIMSWISS population traces follow quite closely those predicted by AIMS, while dTSH starts to deviate at early times, but still falls within the AIMS standard error. AIMSWISS offers accuracy and the possibility of measuring quantities unaccessible to TSH, but for large/challenging systems, where and how would someone establish the compromise between accuracy and speed, that is, when AIMSWISS would be a preferable method over decoherence corrected independent trajectory approaches (TSH, Ehrenfest) to simulate the dynamics of a photoexcited system?
We are of the opinion that independent trajectory approaches which the Reviewer alludes to play a key role in the simulation of photoexcited molecules, and we do not expect AIMSWISS to replace these methods in the near future. On the contrary, we see AIMSWISS as a complementary method that is perfectly placed to assess the quality of a TSH (or Ehrenfest) dynamics via a benchmark. If the methods agree, TSH can be safely used, and if there are discrepancies AIMSWISS – or then OSSAIMS and even AIMS if needed – should be employed. Such an essential benchmark tool for mixed quantum/classical methods has been severely lacking in the toolkit of computational photochemistry until now.

We also fixed a few typographical errors and the style of our references in the main text and the SI based on the Editorial Comments.

We hope that the revised version of our manuscript is now suitable for publication in the Journal of Physical Chemistry Letters.

Sincerely,

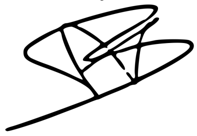

Basile F. E. Curchod
